# Internet-based cognitive behavioural therapy in the real world: Naturalistic use and effectiveness of an evidence-based platform in New Zealand

**DOI:** 10.1177/00048674231183641

**Published:** 2023-06-27

**Authors:** Hayley Guiney, Alison Mahoney, Anna Elders, Charlie David, Richie Poulton

**Affiliations:** 1 Dunedin Multidisciplinary Health & Development Research Unit, Department of Psychology, University of Otago, Dunedin, New Zealand; 2 Clinical Research Unit for Anxiety & Depression, St Vincent’s Hospital, Darlinghurst, NSW, Australia; 3 School of Psychiatry, Faculty of Medicine, University of New South Wales, Sydney, NSW, Australia; 4 Mental Health Solutions, Wise Group, Hamilton, New Zealand

**Keywords:** Digital mental health service, online, cognitive behavioural therapy, anxiety, depression

## Abstract

**Objective:**

Internet-based cognitive behavioural therapy (iCBT) is an efficacious, scalable intervention that could help meet the significant demand for psychological treatment. Yet, there is limited real-world evidence for its effectiveness. This study investigated the use and effectiveness of a free iCBT programme (‘Just a Thought’) in New Zealand.

**Methods:**

We analysed 18 months of user data from the Just a Thought website to understand the characteristics of those who used the Depression and Generalised Anxiety Disorder courses, how many lessons they completed, how mental distress changed across each course and the factors associated with adherence and improvement in mental health.

**Results:**

The results for both courses followed very similar patterns. Course adherence was low overall. There were small differences in adherence by age, gender and ethnicity, and larger differences for those who were ‘prescribed’ Just a Thought by a healthcare worker. Mixed models showed significant reductions in mental distress, with some tapering of improvement across latter lessons. Those most likely to show clinically meaningful reductions in mental distress had completed more lessons, were older and had a higher baseline level of distress.

**Conclusion:**

Alongside previous efficacy research, this real-world data indicate that iCBT is most likely to be effective at the population level and across different subgroups if users complete as much of the course as possible. Strategies to increase course adherence and maximise the public health benefits of iCBT include healthcare workers ‘prescribing’ iCBT and tailored solutions to meet the needs of young people, Māori and Pasifika.

Mental disorders are common but access to evidence-based psychological treatment is limited. Population-based surveys in Australasia indicate that around 20% of people aged 16 years and over meet criteria for a mental disorder in a particular year (Andrews et al., 2001; Wells et al., 2006). Prospective longitudinal studies following the same individuals across their lifecourse show that meeting criteria for a mental disorder at some point in one’s lifetime is much more common than not. For example, in both the Christchurch and Dunedin Health and Development Studies, around 85% of study members had met criteria for at least one mental disorder by the time they reached middle age (Caspi et al., 2020; Schaefer et al., 2017). Given the prevalence of mental disorders, demand for services is high and the degree of unmet need, particularly among those experiencing mild to moderate distress, is significant (Government Inquiry into Mental Health and Addiction, 2018).

To meet this need and ease the associated individual, community and public health burden (GBD 2019 Mental Disorders Collaborators, 2022; Scott et al., 2016), scalable interventions that are effective and accessible for the populations they serve are essential (National Health Service, 2019). Internet-based cognitive behavioural therapy (iCBT) is one such intervention that has the potential to provide faster, more flexible, cost-effective access to evidence-based psychological treatment (Baumann et al., 2020). A large body of research, including meta-analyses and systematic reviews of randomised controlled trials (RCTs), shows that iCBT is an acceptable and efficacious treatment for mild to moderate mental distress (Andrews et al., 2018; Etzelmueller et al., 2020; Karyotaki et al., 2021). Emerging evidence also indicates that iCBT can support people with moderate to severe distress when guided support is provided (Karyotaki et al., 2021).

Despite the potential of iCBT as a population-level intervention, few studies have examined its use and effectiveness in the real world. That is, how the public use, and what mental health benefits are associated with, freely available iCBT tools. Much of the evidence for the benefits of iCBT is from RCTs that provide valuable information about causality but typically prioritise methodological constraints over the diverse needs of the populations the interventions are intended to serve, resulting in the exclusion of large proportions of the population and limited insight into a treatment’s true effectiveness (Tan et al., 2022). People might also engage differently with iCBT programmes when completing them in a self-directed way versus in a trial or research setting (Fleming et al., 2018). However, by 2018, only 11 studies examining real-world use of seven freely available, self-help iCBT programmes had been published (for a review, see the study by Fleming et al., 2018). Across those studies, there was wide variation in the measures used, and few directly reported completion rates. Other studies have examined naturalistic use of iCBT, but those were conducted in a primary care setting with a select group of participants who had sought help for mental distress and been prescribed iCBT by their healthcare provider (Mewton et al., 2013; Newby et al., 2017). Thus, with limited evidence for the uptake and effectiveness of iCBT in the ‘real world’, we do not yet know to what degree it might be a useful population-level intervention.

The purpose of this study was to address these knowledge gaps by providing information about the use and effectiveness of iCBT in the real world by analysing routinely collected data from the New Zealand-based iCBT programme Just a Thought (JaT). Following the launch of JaT in 2019, we analysed 18 months of user data for the generalised anxiety disorder and depression courses (henceforth referred to as the ‘anxiety course’ and ‘depression course’), the most commonly used JaT courses, and the most common mental disorders (GBD 2019 Mental Disorders Collaborators, 2022). We aimed to understand the characteristics of people who used each course, how many lessons they completed, how mental distress changed with course engagement, and the factors associated with greater adherence and improvement over time.

## Method

### Just a thought

JaT (see www.justathought.co.nz) is a free iCBT tool based on THIS WAY UP (TWU; see www.thiswayup.org.au), which was designed and evaluated by clinicians and researchers in Australia (see https://crufad.org/our-research/). A New Zealand-based charitable company (the Wise Group) purchased the licence for the depression and anxiety courses analysed in this article and made minor adjustments to the vocabulary used and the ‘look and feel’ of the courses to ensure accessibility and appropriateness in New Zealand, while retaining the evidence-based CBT content (for more detail, see the study by Mahoney et al., 2021). Each course has six parts (‘lessons’) to it, which use illustrated stories of characters who are experiencing mental distress. The stories teach the psychoeducational and skill-based CBT content to the user. Each lesson is supported by worksheets, which guide the user to apply what they have learned to their own challenges through written exercises and cognitive and behavioural change activities. At the start of each lesson, users complete the Kessler psychological distress scale-10 (K10; Kessler et al., 2002).

### Study design and population

The data analysed here were routinely collected via the JaT website over an 18-month period from 4 April 2020 to 1 November 2021. The depression and anxiety courses were launched in 2019, but we restricted our analyses to 4 April 2020 onwards, when an artificial restriction preventing users from commencing subsequent lessons within 1 week of completing a previous lesson was removed. Over the 18-month period, 14,331 people registered for the anxiety course and 7309 for the depression course. Of those, 9893 (69%) anxiety and 4951 (68%) depression course registrants completed the K10 at the beginning of lesson one and were counted as course commencers. All registrants consented to the collection and use of their non-identifiable data for research, evaluation, and quality improvement purposes by agreeing at registration to the JaT Privacy Policy.

### Variables assessed

#### Age

Registrants provided their year of birth; age in years was estimated from the difference between the year they registered and their reported year of birth. For analysis, people were grouped into four age categories representing relevant developmental epochs: 12 to 24 years (young adulthood), 25 to 44 years (adulthood), 45 to 64 years (middle age) and 65 years and over (older adulthood).

#### Gender

Registrants indicated the gender they identify with as male, female, gender diverse or transgender. To allow sufficient sample size for analysis, gender diverse and transgender users were combined into one category.

#### Ethnicity

Registrants were presented with a list of ethnicities based on the Ethnicity New Zealand Standard Classification 2005 (Statistics New Zealand, 2005), and asked to indicate the ethnicity they most identified with. For analysis, registrants were categorised as Māori, Pacific or European/Other.

#### Source

Registrants came to JaT on their own (self-directed) or after being prescribed the programme by a healthcare worker. Table S1 summarises the prescriptions made by specific professions.

#### Number of lessons completed

Registrants were counted as having completed a lesson if they had clicked a button to load the lesson worksheet, which triggered the website to record a lesson completion date.

#### Mental distress

Registrants completed the K10 at the start of each lesson; scores could range from 10 to 50. The K10 has been shown to be reliable and sensitive to change (Kessler et al., 2002; Merson et al., 2021; Perini et al., 2006). Consistent with previous research, K10 scores between 10 and 15 indicated low mental distress, 16 to 21 moderate distress, 22 to 29 high distress, and 30 to 50 very high distress (Andrews and Slade, 2001).

### Data analysis

All analyses were conducted separately for the anxiety and depression courses. We used descriptive statistics to calculate participant characteristics and lesson completion rates among course commencers (those who completed the K10 at the beginning of lesson one). We focused on course commencers to examine use patterns relevant to those who showed at least minimal engagement with each course, and to enable analysis of change in mental distress with course use (see Table S2 for a comparison of those who stopped after registration versus course commencers). To identify the factors associated with completing more lessons, we used Poisson regression with robust standard errors, the results of which can be interpreted as relative risks. Univariate regressions included each factor on its own, and multivariable regressions included all factors of interest (age, gender, ethnicity, referral source and baseline mental distress) to understand the effect of each factor after adjusting for all others. We then used mixed models with a restricted maximum likelihood estimator to obtain unbiased estimates of mean change in K10 score across the six lessons, with lesson number as a fixed factor, random intercepts and slopes for each participant, and an unstructured covariance matrix. Further sensitivity analyses examined the stability of those findings (see Supplemental Materials).

To investigate patterns of clinically meaningful change with course use, we used an easily interpretable and ecologically valid indicator: course commencers moving from a higher to lower category of distress over their engagement in a JaT course (e.g. from very high to high, moderate or low; from high to moderate or low; or from moderate to low). To identify the factors associated with showing a clinically meaningful decrease across course use, we used Poisson regression with robust standard errors. Relative risks were preferred over odds ratios for our analyses because odds ratios can overestimate effect sizes when the prevalence of the outcome is common (Cummings, 2009). Univariate regressions included each factor on its own, and multivariable regressions included all factors of interest (number of lessons completed, age, gender, ethnicity, referral source and baseline mental distress). Our clinically meaningful change analyses focused on the subset of course commencers who could have shown such change (i.e. completed at least two lessons and had moderate, high or very high baseline mental distress), but note that sensitivity analyses showed comparable patterns of effects in terms of the factors associated with change when all course commencers were included in the denominator (data not shown).

## Results

### Participant characteristics

Table 1 shows the characteristics of people who commenced the anxiety and depression courses in the study period. In both courses, the highest proportion of commencers were aged 25 to 45 years, identified as female, identified as European/Other ethnicity, were self-directed to JaT and had very high baseline K10 scores.

**Table 1.** Characteristics of participants who commenced each course.

	Anxiety course		Depression course	
	*N*	%	*N*	%
Overall	9893		4951	
Age (years)				
12 to 24	2085	22	1308	27
25 to 44	4869	51	2237	46
45 to 64	2314	24	1135	24
65 and over	358	4	143	3
Gender				
Male	2042	21	1138	23
Female	7657	78	3665	75
Gender diverse or transgender	83	1	81	2
Ethnicity				
Māori	722	7	446	9
Pacific	234	2	170	3
European/Other	8937	90	4333	88
Source				
Self-directed	9196	93	4613	93
Prescribed	697	7	338	7
Baseline mental distress				
Low	376	4	93	2
Moderate	1479	15	339	7
High	3452	35	1192	24
Very high	4586	46	3327	67

### Lesson completion rates and associated factors

Table 2 shows the number of lessons (out of six) course commencers completed before ceasing engagement. Lesson completion rates were low in both courses: 73% of anxiety and 69% of depression course commencers had ceased engaging by the end of lesson one. Few completed all six lessons (4% in the anxiety course; 5% in the depression course).

**Table 2.** Lesson cessation points in the anxiety and depression courses, among course commencers.

Stopped after completing lesson	Anxiety course			Depression course		
	*N*	%	Cumulative attrition (%)^a^	*N*	%	Cumulative attrition (%)^a^
0	2810	28	28	961	19	19
1	4456	45	73	2494	50	69
2	1223	12	85	713	14	83
3	562	6	91	305	6	89
4	320	3	94	148	3	92
5	175	2	96	90	2	94
6	347	4	100	240	5	100

a Cumulative percentage of participants who ceased engaging with each course after completing that lesson.

Table 3 shows the results of the univariate and multivariable Poisson regressions used to identify the factors associated with completing more lessons. In both courses and in both regression models, course commencers more likely to complete more lessons were: older (adult, middle-aged and older adults versus young adults); male (versus female); European/Other (versus Māori or Pacific); and prescribed JaT (versus self-directed). There was some evidence that those who identified as gender diverse or transgender completed fewer lessons (versus males), but this effect was not statistically significant in the multivariable analyses, likely due to the small sample size in that subgroup. There were no significant differences by baseline mental distress.

**Table 3.** Factors associated with completing more lessons.

	Anxiety course		Depression course	
	Univariate	Multivariable^a^	Univariate	Multivariable^a^
	RR (95% CI)	RR (95% CI)	RR (95% CI)	RR (95% CI)
Age (years)				
12 to 24	Ref	Ref	Ref	Ref
	–	–	–	–
25 to 44	1.13***	1.13***	1.12**	1.11**
	(1.07, 1.19)	(1.07, 1.20)	(1.05, 1.19)	(1.04, 1.19)
45 to 64	1.13***	1.14***	1.18***	1.16***
	(1.06, 1.21)	(1.07, 1.22)	(1.10, 1.28)	(1.07, 1.25)
65 and over	1.20**	1.18*	1.21*	1.19^†^
	(1.06, 1.36)	(1.04, 1.33)	(1.02, 1.44)	(1.00, 1.42)
Gender				
Male	Ref	Ref	Ref	Ref
	–	–	–	–
Female	0.91***	0.92**	0.86***	0.86***
	(0.86, 0.96)	(0.88, 0.97)	(0.81, 0.91)	(0.81, 0.92)
Gender diverse or transgender	0.77*	0.80	0.82^†^	0.86
	(0.62, 0.97)	(0.64, 1.02)	(0.67, 1.01)	(0.70, 1.06)
Ethnicity				
Māori	0.72***	0.75***	0.78***	0.80***
	(0.66, 0.79)	(0.68, 0.82)	(0.70, 0.87)	(0.72, 0.89)
Pacific	0.84*	0.84*	0.70***	0.72**
	(0.71, 0.99)	(0.71, 0.98)	(0.59, 0.84)	(0.60, 0.87)
European/Other	Ref	Ref	Ref	Ref
	–	–	–	–
Source				
Self-directed	Ref	Ref	Ref	Ref
	–	–	–	–
Prescribed	1.64***	1.67***	1.46***	1.47***
	(1.53, 1.76)	(1.56, 1.79)	(1.33, 1.61)	(1.34, 1.62)
Baseline mental distress				
Low	Ref	Ref	Ref	Ref
	–	–	–	–
Moderate	1.11	1.08	0.90	0.86
	(0.97, 1.29)	(0.94, 1.25)	(0.69, 1.17)	(0.66, 1.13)
High	1.11	1.09	0.87	0.84
	(0.97, 1.27)	(0.95, 1.25)	(0.68, 1.11)	(0.65, 1.08)
Very high	1.02	1.02	0.84	0.85
	(0.89, 1.17)	(0.89, 1.17)	(0.66, 1.06)	(0.67, 1.09)

RR: relative risk; CI: confidence interval.

a Multivariable model includes age, gender, ethnicity, source and baseline mental distress group (low K10 score = 10–15; moderate = 16–21; high = 22–29; very high = 30–50).

*** *p* < 0.001, **p < 0.01, *p < 0.05, ^†^*p* < 0.06.

**Figure 1.**
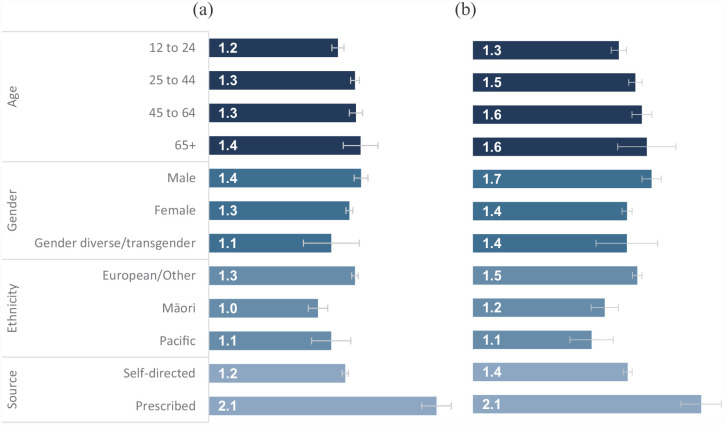
Visualisation of the factors associated with completing more lessons in each course, after adjusting for all others. (a) Anxiety course and (b) depression course. Denominator = course commencers. Bars show the estimated mean number of lessons completed; error bars show 95% confidence intervals. Estimates derived from a multivariable model that included age, gender, ethnicity, source and baseline mental distress group (low K10 score = 10–15; moderate = 16–21; high = 22–29; very high = 30–50).

To illustrate the significant subgroup differences, Figure 1 shows the estimated mean number of lessons completed by each subgroup, based on the multivariable model including age, gender, ethnicity, referral source and baseline K10 score. Figure 1 shows that the observed age, gender and ethnicity differences were small, whereas the difference by referral source was more marked.

### Change in mental distress over time and associated factors

**Figure 2.**
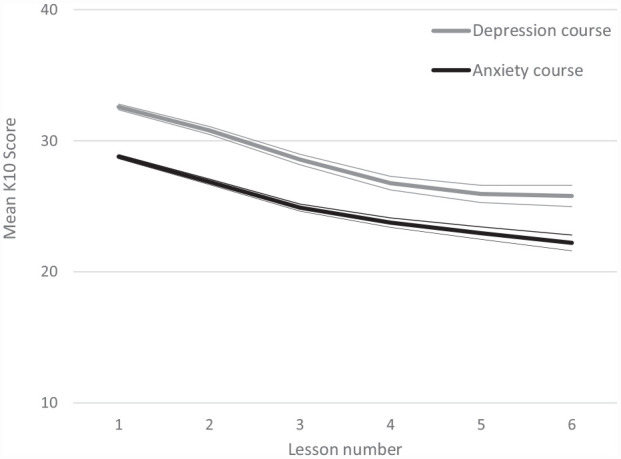
Estimated mean K10 score and associated 95% confidence intervals at each of the six lessons in each course. Participants completed the K10 at the start of each lesson. Estimates derived from a generalised linear mixed model. See Table S2 in the Supplemental Materials for the model parameters.

For each course, Figure 2 shows the estimated mean change in K10 score over the six lessons, derived from a mixed model with lesson number as a fixed factor and random intercepts and slopes for each participant. Recall that people completed the K10 at the start of each lesson. Baseline scores were higher in the depression course, but the rate of improvement across assessment points was similar in both courses. Across the six lessons, course commencers showed statistically significant decreases in distress from baseline (see Table S3 for the specific model parameters). Course commencers tended to show greater improvements across the first three lessons than in the latter ones.

However, given the attrition from one lesson to another, it is possible that the estimated mean change in K10 score over the six lessons provides a misleading picture. To address these concerns, we conducted two sensitivity analyses. First, given that baseline K10 scores differed by age, gender and ethnicity in both courses, and by source in the anxiety course only (data not shown), we repeated the mixed models with age, gender, ethnicity and source as additional fixed factors. The results showed the same pattern of effects for mental distress scores from one lesson to another (see Tables S3 and S4). Second, we considered within-group change in mental distress scores by separating course commencers into groups based on how many lessons they completed. Figure S1 shows that the pattern of within-group change in distress from one lesson to another was consistent in both courses.

**Figure 3.**
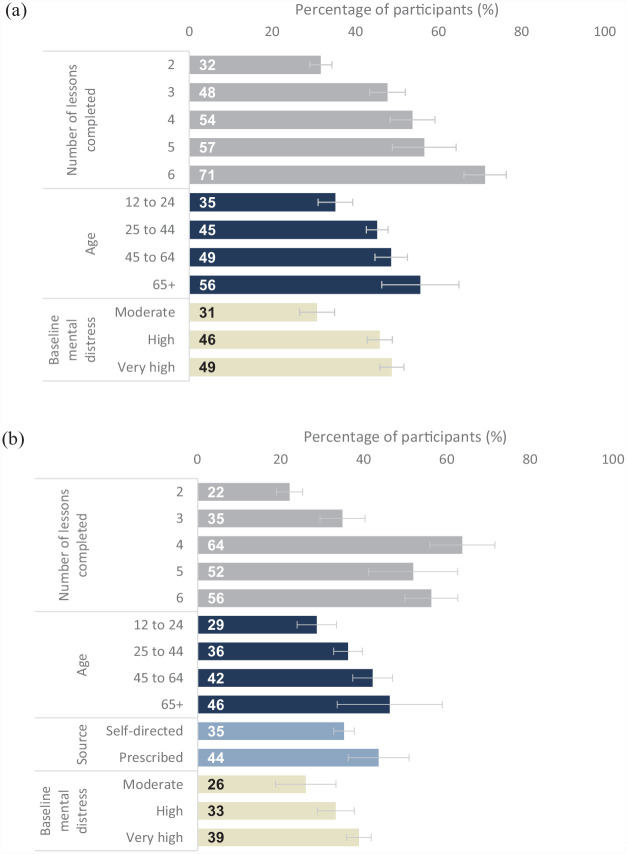
Visualisation of the factors associated with a clinically meaningful decrease in mental distress in each course. (a) Anxiety course and (b) depression course. Denominator = those who could have shown a clinically meaningful change (i.e. had a moderate, high or very high mental distress score at baseline and completed two or more lessons). This group comprised 26% of anxiety course commencers and 30% of depression course commencers. Bars show the estimated percentage of participants in each subgroup who showed a clinically significant improvement from baseline to the last mental assessment they completed. Estimates and 95% confidence intervals are from a multivariable model that included number of lessons completed, age, gender, ethnicity, source and baseline mental distress group (moderate K10 score = 16–21; high = 22–29; very high = 30–50).

A minority (26% of anxiety course commencers, *n* = 2538; 30% of depression course commencers, *n* = 1466) could have shown a clinically meaningful change during their engagement with the course because they had a moderate, high or very high mental distress score at baseline and completed two or more lessons. Among those users, 45% of those in the anxiety course (equivalent to 12% of all course commencers) and 36% of those in the depression course (equivalent to 11% of all course commencers) showed a clinically meaningful reduction in distress from baseline to the last assessment they completed. Table 4 shows the results of the regressions used to identify the factors associated with clinically meaningful reductions in distress. In the multivariable analyses for both courses, the likelihood of showing such a reduction increased with the number of lessons completed, age, and baseline distress. For example, in the depression course, those who completed six lessons were 2.54 (95% CI = [2.12, 3.05]) times more likely than those who completed two lessons to have shown a clinically meaningful decrease in mental distress after adjusting for age, gender, ethnicity, source and baseline mental distress (see Table 4). In addition, in the depression course only, those who were prescribed JaT were more likely than self-directed users to show a clinically meaningful improvement. Figure 3 illustrates the statistically significant effects from the multivariable model.

**Table 4.** Factors associated with a clinically meaningful decrease in mental distress.

	Anxiety course		Depression course	
	Univariate	Multivariable^a^	Univariate	Multivariable^a^
	RR (95% CI)	RR (95% CI)	RR (95% CI)	RR (95% CI)
Number of lessons completed				
2	Ref	Ref	Ref	Ref
	–	–	–	–
3	1.48***	1.51***	1.64***	1.58***
	(1.31, 1.67)	(1.33, 1.70)	(1.33, 2.01)	(1.27, 1.95)
4	1.73***	1.70***	2.92***	2.88***
	(1.52, 1.98)	(1.49, 1.93)	(2.42, 3.52)	(2.38, 3.48)
5	1.76***	1.79***	2.44***	2.34***
	(1.50, 2.06)	(1.52, 2.10)	(1.92, 3.11)	(1.82, 3.01)
6	2.23***	2.25***	2.68***	2.54***
	(2.00, 2.48)	(2.01, 2.51)	(2.24, 3.20)	(2.12, 3.05)
Age (years)				
12 to 24	Ref	Ref	Ref	Ref
	–	–	–	–
25 to 44	1.30***	1.29***	1.35**	1.26*
	(1.13, 1.48)	(1.13, 1.47)	(1.11, 1.64)	(1.04, 1.53)
45 to 64	1.36***	1.38***	1.53***	1.47***
	(1.17, 1.57)	(1.20, 1.60)	(1.25, 1.89)	(1.20, 1.80)
65 and over	1.51***	1.58***	1.84***	1.61**
	(1.22, 1.86)	(1.29, 1.94)	(1.31, 2.58)	(1.17, 2.22)
Gender				
Male	Ref	Ref	Ref	Ref
	–	–	–	–
Female	0.99	1.01	0.85*	0.91
	(0.89, 1.10)	(0.91, 1.12)	(0.73, 0.98)	(0.79, 1.04)
Gender diverse or transgender	0.70	0.87	0.64	0.68
	(0.36, 1.37)	(0.47, 1.58)	(0.30, 1.38)	(0.32, 1.42)
Ethnicity				
Māori	0.98	1.02	0.89	0.93
	(0.80, 1.20)	(0.85, 1.24)	(0.67, 1.19)	(0.69, 1.24)
Pacific	1.04	1.03	0.68	0.77
	(0.77, 1.39)	(0.78, 1.37)	(0.36, 1.30)	(0.39, 1.52)
European/Other	Ref	Ref	Ref	Ref
	–	–	–	–
Source				
Self-directed	Ref	Ref	Ref	Ref
	–	–	–	–
Prescribed	1.09	1.04	1.28**	1.24*
	(0.96, 1.23)	(0.92, 1.17)	(1.07, 1.54)	(1.03, 1.48)
Baseline mental distress				
Moderate	Ref	Ref	Ref	Ref
	–	–	–	–
High	1.43***	1.49***	1.20	1.28
	(1.23, 1.67)	(1.28, 1.73)	(0.86, 1.67)	(0.94, 1.73)
Very high	1.43***	1.58***	1.26	1.49**
	(1.23, 1.66)	(1.36, 1.84)	(0.93, 1.73)	(1.12, 1.99)

RR: relative risk; CI: confidence interval.

Denominator = those who had a moderate, high or very high mental distress score at baseline and completed two or more lessons.

a Multivariable model includes number of lessons completed, age, gender, ethnicity, source and baseline mental distress group (moderate K10 score = 16–21; high = 22–29; very high = 30–50).

*** *p* < 0.001, **p < 0.01, *p < 0.05.

In both courses, there were no statistically significant differences by gender or ethnicity in the proportion who showed clinically meaningful improvements. However, in the depression course, there was some indication of a lower likelihood of meaningful reductions in distress among those who identified as gender diverse/transgender or Pacific, but the low sample sizes in those subgroups meant that those patterns were not statistically significant.

## Discussion

This study assessed the real-world use and effectiveness of iCBT courses for anxiety and depression, which are freely available via the JaT website to the New Zealand public. The patterns observed across the two courses were remarkably similar. Approximately one-half of those who commenced each course completed only one out of six lessons. One-third completed two or more lessons, but only around 5% completed all six lessons. Nevertheless, for the minority of course commencers who persisted through the lessons, meaningful benefits accrued. The largest reductions in mental distress occurred across the first few lessons, and of the minority who could have shown a clinically meaningful improvement (completed two or more lessons and were not in the lowest distress group to begin with), one-third (depression course, 36%; equivalent to 11% of all course commencers) to one-half (anxiety course, 45%; equivalent to 12% of all course commencers) showed meaningful reductions in distress across their engagement in JaT. Together, the findings in this study provide useful insight into who in the New Zealand context is most likely to engage with a newly introduced iCBT programme like JaT, how far through they get, and what factors are associated with greater reductions in mental distress.

### Course commencers

In both courses, the most common commencers were 25 to 44 years old, identified as female and European/Other ethnicity, were self-directed to JaT and had very high levels of mental distress at baseline. Up-to-date nationally representative estimates of the composition of the population experiencing mental distress are not available, but examination of the patterns of mental disorder prevalence highlighted in earlier New Zealand population-based surveys (Wells et al., 2006) suggests that JaT course commencers were likely under-representative of young people, gender diverse or transgender people, Māori and Pasifika. Given high levels of mental distress among young people (Wells et al., 2006) and the common characterisation of young adults as ‘digital natives’ (Prensky, 2001), it is perhaps surprising that they do not represent a greater proportion of JaT course commencers. In contrast, it is not surprising that the majority of course commencers were female and of European descent, given well-established findings that females are more likely than males to seek help for mental distress (Liddon et al., 2018) and that, for systemic and sociological reasons, Māori and Pasifika are less likely to access mental health services (Government Inquiry into Mental Health and Addiction, 2018). Future work, including culturally appropriate consumer and co-design research, is needed to understand how JaT promotion and content can be refined to better appeal to young people, males, gender diverse or transgender people, Māori and Pasifika.

Despite JaT being designed to meet the needs of people experiencing mild to moderate mental distress, those users were in the minority. A high proportion of anxiety (46%) and depression (67%) course commencers had very high mental distress at baseline. This pattern likely reflects the relatively few barriers to seeking out and accessing iCBT for some target groups, compared to those associated with more tailored and intensive face-to-face services. Although risk management strategies can lead iCBT providers to refer severely distressed individuals away from online assistance and towards more intensive and tailored support, the current findings show that iCBT could play a role in assisting those individuals. In both courses, those who had the highest levels of distress at baseline were most likely to show clinically meaningful improvements across their use of the course. Nonetheless, self-directed iCBT is not a panacea: in a recent meta-analysis of RCTs, iCBT was most likely to be beneficial for severely distressed individuals when guided by a healthcare provider (Karyotaki et al., 2021). Blended models of care that integrate digital and in-person treatments are most likely to benefit people in need of mental health services by leveraging the unique benefits of each approach (Cornish, 2020), but more research is needed to inform the effective development and implementation of such models in routine healthcare settings.

### Course adherence and associated factors

Lesson completion rates were low but nevertheless appeared to be comparable to or higher than those seen in other self-directed iCBT programmes (although specific comparisons are not possible due to wide variation in the definition and reporting of course engagement and completion rates; Fleming et al., 2018). Low course adherence is a common finding in ‘real-world’ iCBT research, but few studies have investigated the factors associated with adherence (Wright et al., 2019). We found small differences in completion rates by age, gender and ethnicity (rates were lowest among young adults, females, gender diverse and transgender individuals, Māori and Pasifika), suggesting that research focused on improving course adherence should seek to understand how best to meet the needs of those groups. Consistent with previous research (Baumeister et al., 2014), there was a more notable difference in completion rates by referral source. Those prescribed JaT by a healthcare worker completed more lessons than those who were self-directed, suggesting that healthcare workers prescribing iCBT could be an important modifiable factor to increase course adherence.

### Changes in mental distress and associated factors

Across both courses, course commencers showed statistically significant reductions in mental distress. Consistent with a recent meta-analysis of RCTs, which showed that iCBT treatment effects increased with completion rates (Wright et al., 2019), the likelihood of meaningful reductions in distress increased with the number of lessons completed. After adjusting for lesson completion and the other factors of interest, young people in both courses were the least likely to show clinically meaningful reductions in distress. Alongside population-level data showing that mental disorders are most prevalent in young people (Wells et al., 2006) and the finding that young people completed fewer lessons, that age group appeared to be at a disadvantage. More targeted research is needed to understand how to better tailor iCBT courses for that age group. In contrast, in the depression course only, those who were prescribed JaT had an advantage: they completed more lessons and even after adjusting for course adherence were more likely to show clinically meaningful reductions in distress across course use.

After adjusting for course adherence and the other factors of interest, Māori and non-Māori were similarly likely to show clinically meaningful reductions in distress across course use. Culturally relevant adaptations to JaT that encourage course use and adherence among Māori could, therefore, help to address mental health inequities. While CBT is a western psychological treatment, researchers have shown that it can be adapted to support positive mental health outcomes among Māori (Bennett et al., 2014, 2016). In the New Zealand context, research conducted in partnership with Māori is needed to understand how to best promote and tailor JaT courses, including the ‘look and feel’ and where, how and with whom the courses are used, to enhance course engagement among Māori.

### Limitations

From this observational study alone, we cannot infer causation. Thus, we cannot conclude that healthcare providers prescribing JaT caused users to complete more lessons, or that engaging with JaT caused the observed reductions in mental distress. It is possible that factors not measured here could account for these findings. For example, healthcare providers may have prescribed JaT to those who were better educated, more motivated or better resourced, and those factors could explain the greater course adherence and reductions in mental distress observed here. Moreover, without a control group, we could not eliminate the possibility that the observed reductions in mental distress were due to regression to the mean. Nonetheless, the real-world findings reported here align with those from RCTs that have consistently shown causal benefits of TWU (Andrews et al., 2018), the Australian iCBT programme on which JaT is based and other guided iCBT programmes (Wright et al., 2019). For example, the 10 TWU RCTs (7 trials of the depression course; 3 of the anxiety course) in the meta-analysis by Andrews et al. (2018) showed medium (*k* = 1) to large (*k* = 9) effects of symptom reduction in the intervention versus control conditions. Finally, it is important to note that due to small sample sizes in some minority groups (e.g. Pasifika; people who identified as gender diverse or transgender), the numerical patterns indicating lower effectiveness of the depression course for those groups were not statistically significant. Future real-world iCBT research would benefit from the intentional recruitment of more people from potentially vulnerable groups to help address questions of equity.

## Conclusion

Translating clinical trial evidence into the real world is a formidable challenge (Marchand et al., 2011). By evaluating iCBT courses for depression and anxiety in the New Zealand context using a large sample of routinely collected user data, this study provides evidence that can be used to guide improvements to the digital tools available to the public and to support a stepped care model in which iCBT could be incorporated into primary care and culturally responsive services. Alongside previous efficacy research, this real-world data indicate that iCBT is most likely to be effective at the population level and across different subgroups if course adherence is maximised. Strategies to increase course adherence may include healthcare workers ‘prescribing’ iCBT and developing tailored solutions to better attract and meet the needs of young people, males, gender diverse or transgender people, Māori and Pasifika.

## Supplemental Material

sj-docx-1-anp-10.1177_00048674231183641 – Supplemental material for Internet-based cognitive behavioural therapy in the real world: Naturalistic use and effectiveness of an evidence-based platform in New Zealand Supplemental material, sj-docx-1-anp-10.1177_00048674231183641 for Internet-based cognitive behavioural therapy in the real world: Naturalistic use and effectiveness of an evidence-based platform in New Zealand by Hayley Guiney, Alison Mahoney, Anna Elders, Charlie David and Richie Poulton in Australian & New Zealand Journal of Psychiatry
