# Supplementary material for: Internet-based cognitive behavioural therapy in the real world: Naturalistic use and effectiveness of an evidence-based platform in New Zealand
Source: Aust N Z J Psychiatry. 2023 Jun 27;58(3):238–49. doi: 10.1177/00048674231183641 (PMC10903121; doi:10.1177/00048674231183641)
Supplement: sj-docx-1-anp-10.1177_00048674231183641 – Supplemental material for Internet-based cognitive behavioural therapy in the real world: Naturalistic use and effectiveness of an evidence-based platform in New Zealand [file sj-docx-1-anp-10.1177_00048674231183641.docx]

**Supplementary Materials**

**Professions of Just a Thought prescribers**

Table S1 shows the specific professions of people who prescribed Just a Thought to the anxiety and depression course users analysed in this study. In both courses, the most common prescribers were general practitioners and nurses or nurse practitioners.

Table S1. *Professions of Just a Thought course prescribers*

|  | Anxiety course | |  | Depression course | |
| --- | --- | --- | --- | --- | --- |
|  | *N* | *%* |  | *N* | *%* |
| General Practitioner | 228 | 33% |  | 106 | 31% |
| Nurse / Nurse Practitioner | 123 | 18% |  | 71 | 21% |
| Psychologist | 101 | 14% |  | 36 | 11% |
| Other Allied Health | 71 | 10% |  | 28 | 8% |
| Counsellor | 59 | 8% |  | 32 | 9% |
| Social Worker | 56 | 8% |  | 27 | 8% |
| Other | 48 | 7% |  | 26 | 8% |
| Other Medical Specialist | 6 | 1% |  | 8 | 2% |
| Psychotherapist | 5 | 1% |  | 4 | 1% |

**Differences between course registrants and course commencers**

Our primary analyses focused on course commencers (those who had completed the K10 at the beginning of lesson one) to examine ‘real world’ use patterns relevant to users who showed at least minimal engagement with the depression and anxiety courses, and to enable analysis of change in mental distress with course use. Here we consider whether the characteristics of those who registered but progressed no further (i.e., did not complete the baseline K10 and therefore had no mental distress score recorded) differed from course commencers. In both courses, approximately one-third (31% in the anxiety course; 32% in the depression course) of users stopped after registration; the remainder were considered course commencers.

Table S2 shows the characteristics of the two user groups: those who stopped after registration and course commencers. In both the anxiety and depression courses, chi-squared analyses indicated different age and gender distributions in the two groups. Compared with those who stopped after registration, course commencers included higher proportions of users younger than 45 years and males. In the anxiety course only, there was a small ethnicity difference, such that course commencers included a slightly lower proportion of Māori than those who stopped after registration. There were no differences by source, indicating similar levels of course commencement among those who were prescribed versus self-directed to Just a Thought.

Consideration of these findings alongside the main analysis of the factors associated with completing more lessons indicates that the patterns of attrition from registration to completing the baseline K10 at the start of lesson one are not always the same as those for persisting through the lessons once started. In both the anxiety and depression courses, older adults showed greater levels of attrition between registration and the beginning of the first lesson, but once they had commenced a course, older adults were more likely than younger adults to complete more lessons. While attrition from registration to the start of lesson one was similar for those who were prescribed versus self-directed to Just a Thought, once a course was commenced, users who were prescribed the course were more likely to complete more lessons. There were also some consistent attrition patterns: females and non-European users were slightly less likely to continue after registration and slightly less likely to persist through the lessons once they had commenced a course.

Table S2. *Characteristics of those who stopped after registration versus course commencers*

|  | Anxiety course | |  | Depression course | |  |
| --- | --- | --- | --- | --- | --- | --- |
|  | Stopped after registration | Course commencers |  | Stopped after registration | Course commencers | |
| Age | *Χ^2^* = 100.79*** | |  | *Χ^2^* = 14.69** | | |
| 12 to 24 years | 19% | 22% |  | 25% | 27% | |
| 25 to 44 years | 45% | 51% |  | 45% | 46% | |
| 45 to 64 years | 30% | 24% |  | 25% | 24% | |
| 65 years and over | 6% | 4% |  | 4% | 3% | |
| Gender | *Χ^2^* = 22.01*** | |  | *Χ^2^* = 9.54** | | |
| Male | 18% | 21% |  | 20% | 23% | |
| Female | 81% | 78% |  | 78% | 75% | |
| Gender diverse or transgender | 1% | 1% |  | 1% | 2% | |
| Ethnicity | *Χ^2^* = 7.05* | |  | *Χ^2^* = 0.60 | | |
| Māori | 9% | 7% |  | 9% | 9% | |
| Pacific | 3% | 2% |  | 3% | 3% | |
| European/Other | 89% | 90% |  | 88% | 88% | |
| Source | *Χ^2^* = 0.25 | |  | *Χ^2^* < 0.01 | | |
| Self-directed | 93% | 93% |  | 93% | 93% | |
| Prescribed | 7% | 7% |  | 7% | 7% | |

****p* < .001, ***p* < .01, **p* < .05

**Generalised linear mixed model parameters and sensitivity analyses**

Table S3 shows the parameters for the generalised linear mixed models used to understand the change in mental distress scores associated with engagement in the anxiety and depression courses. Recall that participants’ mental distress was assessed by the K10 at the start of each lesson. On average, by the start of lesson 3 in both courses, participants’ K10 scores had reduced by four points. By the start of lesson 6 in both courses, participants’ scores had reduced by an average of approximately seven points.

Table S3. *Model parameters for the generalised linear mixed models assessing change in mental distress across use of the Just a Thought anxiety and depression courses*

|  | Anxiety course | |  | Depression course | |
| --- | --- | --- | --- | --- | --- |
| Fixed effects | *Β* | 95% CI |  | *Β* | 95% CI |
| Lesson 1 | Ref |  |  | Ref |  |
| Lesson 2 | -1.9 | (-2.1, -1.7)*** |  | -1.8 | (-2.0, -1.5)*** |
| Lesson 3 | -3.9 | (-4.1, -3.6)*** |  | -4.0 | (-4.4, -3.6)*** |
| Lesson 4 | -5.0 | (-5.4, -4.7)*** |  | -5.8 | (-6.3, -5.3)*** |
| Lesson 5 | -5.8 | (-6.3, -5.4)*** |  | -6.6 | (-7.3, -6.0)*** |
| Lesson 6 | -6.6 | (-7.2, -6.0)*** |  | -6.8 | (-7.6, -6.0)*** |
|  |  |  |  |  |  |
| Random effects | Variance | 95% CI |  | Variance | 95% CI |
| Individual | 52.6 | (50.1, 55.3) |  | 49.1 | (45.8, 52.7) |
| Time | 1.8 | (1.5, 2.1) |  | 1.9 | (1.6, 2.4) |
| Individual, time covariance | -2.8 | (-3.7, -2.0) |  | -2.0 | (-3.1, -0.9) |
| Residual | 12.3 | (11.8, 12.8) |  | 13.1 | (12.4, 13.9) |

^1^Each model included lesson number as a fixed factor and random intercepts and slopes for each participant. ****p* < .001

Given that baseline K10 scores differed by age, gender, and ethnicity in both the anxiety and depression courses, and by source in the anxiety course only (data not shown), we repeated the mixed models with age, gender, ethnicity, and source as additional fixed factors. The results showed the same pattern of change in mental distress from one lesson to the next (see Table S4).

Table S4. *Model parameters for the generalised linear mixed models assessing change in mental distress across use of the Just a Thought anxiety and depression courses after adjusting for age, gender, ethnicity and source*

|  | Anxiety course | |  | Depression course | |
| --- | --- | --- | --- | --- | --- |
| Fixed effects | *Β* | 95% CI |  | *Β* | 95% CI |
| Lesson number |  |  |  |  |  |
| Lesson 1 | Ref |  |  | Ref |  |
| Lesson 2 | -1.9 | (-2.1, -1.7)*** |  | -1.7 | (-2.0, -1.5)*** |
| Lesson 3 | -3.9 | (-4.1, -3.6)*** |  | -3.9 | (-4.3, -3.6)*** |
| Lesson 4 | -5.0 | (-5.3, -4.6)*** |  | -5.7 | (-6.2, -5.2)*** |
| Lesson 5 | -5.8 | (-6.3, -5.3)*** |  | -6.6 | (-7.2, -5.9)*** |
| Lesson 6 | -6.6 | (-7.2, -6.0)*** |  | -6.7 | (-7.5, -5.9)*** |
| Age group |  |  |  |  |  |
| 12 to 24 years | Ref |  |  | Ref |  |
| 25 to 44 years | -3.5 | (-3.8, -3.1)*** |  | -4.0 | (-4.5, -3.5)*** |
| 45 to 64 years | -5.9 | (-6.3, -5.4)*** |  | -6.2 | (-6.7, -5.6)*** |
| 65 years and over | -7.3 | (-8.1, -6.5)*** |  | -8.5 | (-9.8, -7.3)*** |
| Gender |  |  |  |  |  |
| Male | Ref |  |  | Ref |  |
| Female | 0.3 | (-0.1, 0.6) |  | 0.4 | (-0.1, 0.9) |
| Gender diverse or transgender | 3.9 | (2.2, 5.5)*** |  | 3.3 | (1.7, 5.0)*** |
| Ethnicity |  |  |  |  |  |
| Māori | 1.4 | (0.9, 2.0)*** |  | 0.8 | (0.1, 1.5)* |
| Pacific | 1.2 | (0.2, 2.1)* |  | 1.2 | (0.0, 2.3)* |
| European/Other | Ref |  |  | Ref |  |
| Source |  |  |  |  |  |
| Self-directed | Ref |  |  | Ref |  |
| Prescribed | 0.3 | (-.3, 0.9) |  | -0.2 | (-1.0, 0.6) |
| Random effects | Variance | 95% CI |  | Variance | 95% CI |
| Individual | 47.5 | (45.1, 50.0) |  | 42.9 | (39.8, 46.3) |
| Time | 1.8 | (1.5, 2.1) |  | 1.9 | (1.6, 2.4) |
| Individual, time covariance | -2.7 | (-3.6, -1.9) |  | -2.2 | (-3.2, -1.1) |
| Residual | 12.1 | (11.6, 12.6) |  | 13.1 | (12.4, 13.9) |

^1^Each model included lesson number as a fixed factor and random intercepts and slopes for each participant. ****p* < .001, **p* < .05

Separating course commencers into groups based on how many lessons they completed allowed us to examine within-group change across the six lessons. As shown in Figure S1, where it was possible to measure, the pattern of within-group change in mental distress from one lesson to another (slope) was consistent in both courses. For example, the slope from lesson one to two was similar for those who stopped after completing two, three, four, five, and six lessons. Based on these analyses and those in the main text showing that baseline mental distress was not related to the number of lessons completed, it appears that discontinuing was not related to the magnitude of change in mental distress.

1. Anxiety course
2. Depression course

*Figure S1.* Observed mean K10 score at each lesson, separated into groups based on the number of lessons participants completed. Confidence intervals are not shown due to a high degree of overlap between the groups. Participants completed the K10 at the start of each lesson.
